# HARNESSING HEALTH SERVICES RESEARCH TO FUEL FRAILTY IMPLEMENTATION AND INNOVATION

**DOI:** 10.1093/geroni/igae098.1118

**Published:** 2024-12-31

**Authors:** Megan Huisingh-Scheetz

**Affiliations:** University of Chicago, Chicago, Illinois, United States

## Abstract

Broad implementation of frailty assessment and management in clinical practice has been a core mission of geriatricians for decades, and now medical and surgical subspecialists have joined the effort. Dr. Terrie Fox Wetle has demonstrated the power of health services research paired with passion to foster change. In this presentation, I will pay tribute to Dr. Wetle, review the health services research and its application to frailty science, and reflect on the importance of passion to one’s career. I will share successes and remaining challenges of frailty implementation as exemplified in the Successful Aging and Frailty Evaluation clinic established in 2011. I will then discuss novel directions for frailty assessment and management using technology that may help overcome hurdles. Throughout the presentation, I will highlight mentors, collaborators and obstacles that have shaped my direction. I will end with a call for team science and policy change to advance frailty care.

